# Epidural Anesthesia for Cesarean Section in a Pregnant Woman with Marfan Syndrome and Dural Ectasia

**DOI:** 10.1155/2017/2126310

**Published:** 2017-05-22

**Authors:** Franco Pepe, Mariagrazia Stracquadanio, Francesco De Luca, Agata Privitera, Elisabetta Sanalitro, Puccio Scarpinati

**Affiliations:** ^1^U.O.C. Ostetricia e Ginecologia e PS, Ospedale Santo Bambino, Catania, Italy; ^2^Istituto di Patologia Ostetrica e Ginecologica, Ospedale Santo Bambino, Catania, Italy; ^3^U.O. Cardiologia Pediatrica, Ospedale Santo Bambino, Catania, Italy; ^4^Modulo Dipartimentale Anestesia Ostetrica, Ospedale Santo Bambino, Catania, Italy

## Abstract

Marfan syndrome (MFS) is a genetic disorder of connective tissue, characterized by variable clinical features and multisystem complications. The anesthetic management during delivery is debated. Regional anesthesia has been used with success during cesarean delivery, but in some MFS patients there is a probability of erratic and inadequate spread of intrathecal local anesthetics as a result of dural ectasia. In these cases, epidural anesthesia may be a particularly useful technique during cesarean delivery because it allows an adequate spread and action of local anesthetic with a controlled onset of anesthesia, analgesia, and sympathetic block and a low risk of perioperative complications. We report the perioperative management of a patient with MFS and dural ectasia who successfully underwent cesarean section using epidural technique anesthesia. The previous pregnancy of this woman ended with cesarean section with a failed spinal anesthesia that was converted to general anesthesia due to unknown dural ectasia at that time.

## 1. Introduction

Marfan syndrome (MFS) is an autosomal dominant hereditary disorder of connective tissue; its incidence is estimated to be around 1 : 5.000, with no differences in gender or ethnic background. In 90% of cases, it is associated with mutations in the FBN1 gene that encodes fibrillin [1]. The clinical manifestations of the gene may involve multiple organs with various severity, particularly affecting the cardiovascular, skeletal, and ocular systems. The clinical and instrumental diagnosis is based on observation of the Ghent criteria, proposed in 1996 by De Paepe et al. [2], ranging from the familiarity to multiorgan involvement and they were recently revised by Loeys et al. in 2010 [3]. Some manifestations are evident since childhood (such as ectopia lentis), while others were at a later date, such as the lumbosacral dural ectasia; the main cause of morbidity and mortality is related to aortic dilation and acute aortic dissection. Cardiovascular manifestations, such as aortic dilatation and dissection, are responsible for 90% of deaths attributed to MFS [4, 5]. The disease is not associated with a reduction in fertility; in fact it is common to find a pregnant woman with the syndrome. In such a case, it would be appropriate to have an accurate clinical evaluation before pregnancy, particularly an echocardiography, to assess the size of the aortic root: a diameter greater than 40 mm puts the patient at risk of its rupture.

As reported in literature, the obstetric management of women with MFS seems now well coded, with favorable outcome if the aortic root diameter is less than 40 mm. The increase in aortic size during pregnancy is not unique in women with MFS but is known to occur during normal healthy pregnancies and with increased severity in women with preeclampsia [6].

Some recent guidelines advise women with MFS to avoid pregnancy or, alternatively, undergo surgical ascending aortic replacement prior to conception, if the aorta measures > 4 cm [7].

Literature suggests a 1% risk of aortic dissection or significant cardiac event in women with an aortic root diameter of <40 mm [8]. The risk is increased when the aortic root diameter is >40 mm, if there is a rapid increase in aortic dimensions or in the context of a family history of dissection [9].

However, the presence of ectasia of the dural sac has been considered the major cause of failure of locoregional anesthesia during cesarean section. The purpose of this study is to present the case of a MFS pregnant woman at term with an extensive dural ectasia who had a successful cesarean section with epidural anesthesia during her second pregnancy.

## 2. Case Report

F. S. is a 35-year-old, 180 cm tall, 85 Kg patient, suffering from MSF with a lumbosacral dural ectasia, who was subjected to a cesarean section at 37 weeks and 3 days of gestation. She reports that the mother was very high and died suddenly before the age of 50; her maternal grandfather was particularly high too, and her brother was myopic and had severe scoliosis. Medical history was positive for ectopia lentis (diagnosed when she was 6 years old), dorsal scoliosis (treated with corset from 10 to 14 years of age), and mild ectasia of the aortic arch. Previously she underwent right saphenectomy and right breast fibroadenoma enucleation. Physical examination showed skeletal abnormalities such as high arched palate, opening of the arms greater than height, pectus carinatum internalized to the right, flat feet, bilateral valgus, and arachnodactyly. Striae were evident on the skin of her chest, shoulders, back, and abdomen. On cardiovascular examination, a metallic click and systolic murmur were auscultated with a stethoscope. Heart sounded valid and rhythmic, with good hemodynamic compensation, and ECG had a normal sinus rhythm and normal track with a medium pulse of 65 beats per minute. The patient was normotensive (BP 120/70 mmHg). Echocardiogram showed a mild dilatation of the aortic root (42 mm), normal ventricular function, and a mild mitral valve prolapse without regurgitation.

The obstetric history showed an uneventful previous C-section delivery in 2004. She had a spinal block anesthesia after spinal anesthesia, which was converted into a general one. In the postoperative period, she had a hemorrhage due to uterine atony treated with oxytocin and prostaglandins and recovery in intensive care. After five days, the patient was discharged in good medical condition and she was followed up every 6 months and had a prophylactic therapy with beta-blockers. In 2007, because of a lumbar pain, she performed a lumbosacral MRI that showed ectasia of the distal dural sac with cystic dilatation of some nerve roots. This finding is one of the major diagnostic criteria of MFS. In 2009, she started a second pregnancy and she was under the care of the outpatient obstetric clinic of the Santo Bambino Hospital in Catania (Sicily). During her first trimester of pregnancy, the patient was asymptomatic with good cardiovascular compensation and she did not take any medication. She has been monthly subjected to obstetric visits; she also had three ultrasound scans (one for each trimester of pregnancy), two cardiological examinations (at the beginning and near term), and two maternal echocardiographies (at 22 weeks and near term) in order to monitor the aortic root. The fetal growth was regular. At 37 weeks + 3 days, because of the occurrence of uterine contractions, the patient was admitted to the hospital for cesarean section. Considering the previous bad experience during spinal anesthesia (lack of efficacy with use of general anesthesia), being aware of the presence of the ectasia of the dural sac during the preoperative evaluation of the patient, epidural anesthesia was proposed to perform her second cesarean section. This type of anesthesia allows a better circulation and distribution of the anesthetic, overcoming the problems related to spinal anesthesia in the presence of dural ectasia. The drugs administered epidurally require dosages from 5 to 10 times higher and volumes greater than those calculated for the subarachnoid space. The advantages of an epidural block include a lower incidence and severity of maternal hypotension, thanks to the reduced rate of sympathetic block, a lower risk of headache due to accidental dural puncture, and the possibility of an accurate control of level and duration of anesthesia. The patient was informed about the type of regional anesthesia chosen and monitored. She was continuously under noninvasive monitoring (ECG, arterial blood pressure, and oxygen saturation) and she was premedicated with ranitidine 50 mg and metoclopramide 10 mg in saline solution. She was placed in a sitting position and then the locoregional block in epidural anesthesia was performed by a midline approach with the placement of an epidural catheter, through a 17-gauge Tuohy needle, positioned between L2 and L3. She was given lidocaine 400 mg (20 ml of 2%) with 1 mEq of sodium bicarbonate and 50 mcg of fentanyl. The anesthetic block was manifested within three minutes without side effects. After 15 minutes, the cesarean section started, because the sensory block was sufficiently high (T4) for cesarean section. The systolic blood pressure remained stable (110–125/70 mmHg) for the entire duration of surgery and the postoperative period. There was no evidence of intraoperative and postoperative complications and the patient did not report any pain symptoms. Short-term prophylaxis for infection was administered (3 g ampicillin/sulbactam) after the delivery of the baby as well as 20 IU of oxytocin. After 30 minutes from the anesthetic block, 1 mg of morphine + 75 mcg of clonidine and 12 mg of naropine were injected in the epidural space through the catheter. After an hour from the beginning of the anesthetic block, an ongoing anesthesia with 0,1% naropine, 250 mg at 10 ml/h, was placed in the infusion pump for epidural. At the end of the surgery, for further analgesia, a 75 mg of diclofenac i.m and 0.2 mg of methylergometrine i.m. were administered. The patient was kept under observation for 2 hours and then transferred to the ward. The male newborn was 3.250 g, and he was extracted after 30 minutes since the moment epidural catheter was placed and 1 minute after the skin incision. The Apgar score in the first minute was 9, and it was 10 after five minutes. The epidural catheter was removed 12 hours after cesarean section. The postoperative course was regular, with her discharge on the fourth day after C-section. A 12-day heparin prophylaxis was performed for venous thromboembolism prevention. After a follow-up of five years, we can assert that the patient is in good health and the aortic root diameter is always 42 mm.

## 3. Discussion

MFS is an autosomal dominant disorder of the connective tissue related to mutation of the gene for fibrillin, a glycoprotein that is the major component of extracellular microfibrils, whose gene maps to chromosome 15. MFS involves different organs and systems with varying severity: for this reason, its diagnosis is mainly clinical and instrumental and, then, molecular. It is based on the observation of the Ghent criteria and revised criteria [10].

In literature, there are many experiences on the management of pregnant women with MS [9–13]. Pregnancy can be considered at low risk in the absence of significant aortic dilatation and mitral insufficiency. In women with low cardiac involvement, the risk of aortic dissection, endocarditis, and congestive heart failure in pregnancy is estimated to be only 1%. The risk is higher during the third trimester of pregnancy due to the increase of the hemodynamic stress. During pregnancy and postpartum period, echocardiography should be frequently performed, depending on the extent of the initial dilatation of the aortic root, in order to monitor the cardiovascular system and the possible progressive aortic dilatation.

Beta-blockers reduce the risk of aortic dilatation and cardiac complications, but they seem to increase the tone and uterine contractility, and they might reduce the flow in the umbilical artery causing low birth weight infants. They were not used in our pregnant woman.

The dural ectasia is one of the major criteria for diagnosis of MFS and it is present in over 2/3 of adults affected [14–16] and the prevalence of severe (degrees 2 and 3) involvement of dura mater was higher in patients harboring premature termination codon mutations compared to those carrying missense mutations [17]. It is hypothesized that, in Marfan's syndrome, the dura mater is weaker and, as a result, cerebrospinal fluid pulsation eventually leads to dural ectasia with gradual bone erosion. The dural ectasia is characterized by a swelling of the dural sac and of the spinal canal and, sometimes, of the nerve sheaths. Although it can occur along the entire channel, the most frequent site is the lumbosacral spine. The most common clinical symptoms, which can be intensified by the supine position, are low back pain, headache, asthenia, decreased sensitivity below and around the affected section, and, occasionally, rectal pain and/or discomfort in the genital area [16, 18]. The extension of the dural expansion is variable; sometimes the lesion is confined to focal dilation of the dural coating of the nerve roots, near their exit from the spinal column: they are called “radicular cysts.” The chronic dilatation of the dural sac can also exert an erosive effect against adjacent bone structures of the spine. The indirect signs of bone damage can be observed with radiographic test (Rx) and by examination of Computed Tomography (CT). However, the gold standard for the evaluation of dural ectasia is RM, for the quality of anatomical detail and for its multiplanarity. The prevalence of dural ectasia in patients with MS is variable from 63% to 92%, probably in relation to the imaging modality used in the various studies [19–21]. In a study published in 1999, out of 83 MFS patients examined by MRI, the dural ectasia was detected in 92% of cases and in none of the patients in the control group. However, high prevalence of dural ectasia (41%) exits even in patients with MFS without back pain [16]. No correlation was found with the presence of aortic dilatation; therefore, dural ectasia has no predictive value on cardiovascular prognosis in these patients. Regarding the clinical expression, dural ectasia is often clinically silent or can be occasionally associated with low back pain or lumbosciatica. However, a clear correlation between low back pain and dural ectasia has not been demonstrated.

For the best management of labor and delivery of MFS patients, it is clear that the primary goal is the reduction of cardiovascular stress, and cesarean section is often performed for the prevention of cardiovascular complications. Patients with an aortic root < 4 cm in diameter at the time of delivery have a similar outcome for vaginal and cesarean section delivery, but cesarean section is preferred in patients with an aortic root > 4 cm because the risk for cardiac decompensation is extremely high [22]. However, aortic dissection has been reported even in the absence of preexisting aortic root dilatation [22].

Fluctuation in hemodynamic parameters secondary to pain and anxiety of labor may have negative effects on the cardiovascular system; high blood pressure tends to develop aortic aneurysms due to weakened vascular media in patients with MFS, and myocardial ischemia and heart failure can also be caused by an increased myocardial oxygen demand resulting in high blood pressure; thus, the main goal is to prevent high blood pressure [23]. For all these reasons, cesarean section is frequently planned.

The type of anesthesia has been discussed too. General anesthesia causes blood pressure variations during intubation; therefore peripheral anesthesia seems preferable because of slow onset and gradual progression of epidural block. Since the spontaneous birth determines increase in blood pressure during contractions, the use of epidural analgesia reduces pain, blood pressure, and heart rate. Cesarean section was performed in our patient because she already had a cesarean section.

In some studies, regional anesthesia has been practiced successfully in MFS patients, both during labor analgesia and during cesarean section. Combined spinal-epidural anesthesia is preferred over general anesthesia for cesarean section in patents with MFS because combined spinal-epidural anesthesia provides excellent hemodynamic stability, and adequate postoperative pain control may be obtained via epidural analgesia. However, many cases of spinal anesthesia failure have been reported in Marfan patients, possibly due to dural ectasia [24, 25]. Few cases of incorrect or inadequate spread of intrathecal local anesthetic in patients with this syndrome have been described. Lacassie et al. [26] performed continuous spinal anesthesia in two patients with an incrementally increased dose of bupivacaine, but they stopped further administration of bupivacaine after 21 ml for the fear of potential neurological damage. They also reported an irregular distribution of spinal anesthesia due to unpredictable and inadequate spread of intrathecal local anesthetics in patients with MFS. One of the most important factors influencing the height of the block in patients receiving spinal anesthesia is the volume of CSF in the lumbosacral space, which contributes to the variability in the spread of spinal block. Kim et al. [27] reported a successful perioperative management of a patient with MFS and dural ectasia for cesarean section using epidural anesthesia. The surgically adequate level of anesthesia was achieved 30 min after the epidural injection of 27 ml of 2% lidocaine with epinephrine (1 : 200) and fentanyl (100 mcg).

In summary, the evaluation of pregnant women with MFS requires multidisciplinary management with a close cooperation between gynecologist, cardiologist, anesthesiologist, and neonatologist. Pregnancy should be programmed after complete evaluation of the patient and the definition of specific risks. Relevant is the echocardiographic assessment of aortic root dilation. During pregnancy, the obstetric management is not significantly different, but it is burdened with a higher frequency of premature rupture of membranes, the side effects of the drugs used, where indicated, for the prevention of aortic rupture, and the risk of aortic dissection. The anesthetic management during delivery is debated. Regional anesthesia has been successfully used during cesarean section, but there is a significant probability of erratic and inadequate intrathecal spread of local anesthetics, most likely as a result of dural ectasia. In these patients, epidural anesthesia may be a particularly useful technique during cesarean delivery because it allows adequate spread and action of local anesthetic and controlled onset of anesthesia, analgesia, and sympathetic block with low risk of complications. We report the perioperative management of a patient with MFS and lumbosacral dural ectasia who underwent successful cesarean delivery using epidural technique anesthesia. In her previous pregnancy, the failed spinal anesthesia during cesarean section was converted to general anesthesia due to the unknown presence of dural ectasia at that time.
